# Limbus Vertebra

**DOI:** 10.7759/cureus.13954

**Published:** 2021-03-17

**Authors:** Sharzad J Alagheband, Adrianna D Clapp, Dusty M Narducci, Ryan Cudahy, George Pujalte

**Affiliations:** 1 Department of Internal Medicine, Winthrop University Hospital, Mineola, USA; 2 Department of Family Medicine, Mayo Clinic, Jacksonville, USA; 3 Department of Family Medicine, University of South Florida, Tampa, USA; 4 Department of Family Medicine and Sports Medicine, Dignity Health Medical Group, San Francisco, USA

**Keywords:** limbus vertebra, back pain, repetitive spinal extension, herniation

## Abstract

Athletes in their teenage years can present to clinics with back pain, without any history of trauma. Many sports require repetitive spinal extension, which may be pertinent to the evaluation of back pain as a chief complaint. Musculoskeletal and neurologic examinations are crucial in the evaluation of athletes presenting with back pain. Most back pain cases are caused by benign conditions that resolve with conservative treatment. However, radiographic imaging may be appropriate to look for possible spondylolysis in teenage athletes who perform repetitive extension in their sports, and who present with a positive stork test on examination. Limbus vertebra is a condition that can be seen in asymptomatic patients but can also be associated with back pain. Nevertheless, a conservative approach is still appropriate in these cases, with escalation to further testing or imaging only considered for recalcitrant pain. Limbus vertebra is not well known by clinicians and can be misdiagnosed. Therefore, early recognition is crucial to potentially prevent an unnecessary cascade of increasing expenses related to time, effort, medications, and resources to find the diagnosis when conservative treatment is preferred.

## Introduction

Limbus vertebra was first described by Christian Schmorl in 1927 as an intraosseous herniation of the nucleus pulposus through the ring apophysis, either anteriorly or posteriorly, and is thought to be a consequence of injury to immature skeleton in a child or an adolescent [1]. Literature has qualified this finding as a normal variant of the spine [2-4]. Many patients with this condition are asymptomatic; however, it can cause both acute or chronic back pain symptoms [5-8]. Limbus vertebra is not well known by physicians and can be misdiagnosed as a fracture, discitis, Schmorl’s node or a tumor, resulting in further unnecessary and even invasive diagnostic procedures [6,9]. A helpful orthopedic maneuver to aid in diagnosis is the stork test for spondylosis, whereby the patient stands on one leg and performs lumbar extension. This is then repeated with the other leg. The test is positive if there is ipsilateral pain during extension, which indicates lumbar joint pathology such as spondylosis, spondylolisthesis, or a limbus vertebra [6-8].

## Case presentation

**Figure 1:**
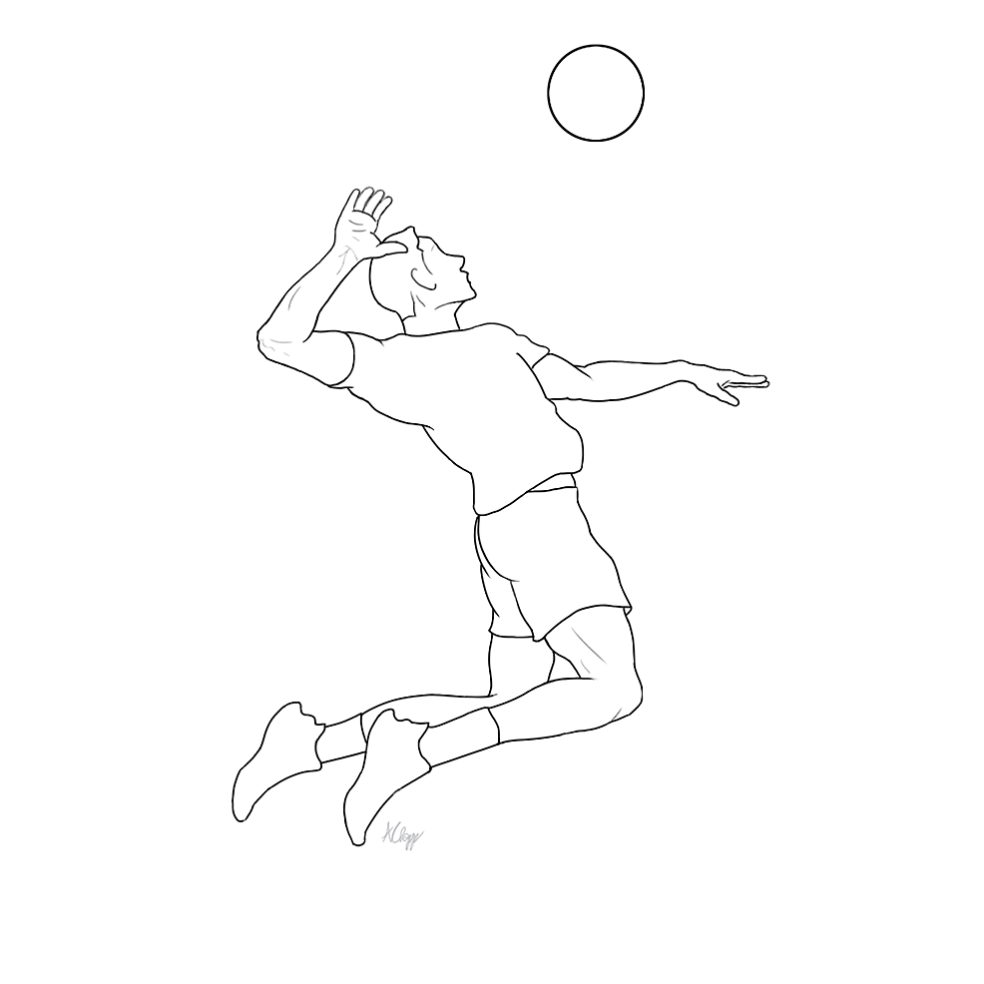
Sports that involve repetitive spinal extension, such as in volleyball with arms overhead during jumping, can cause lower right back pain and pathology. The figure is original and was designed for this article.

An 18-year-old male volleyball player presented to the clinic with a three-week history of lower back pain. The patient was otherwise healthy, with no history of trauma. Spinal extension with his arms overhead during jumping elicited lower right back pain (Figure 1).

**Figure 2:**
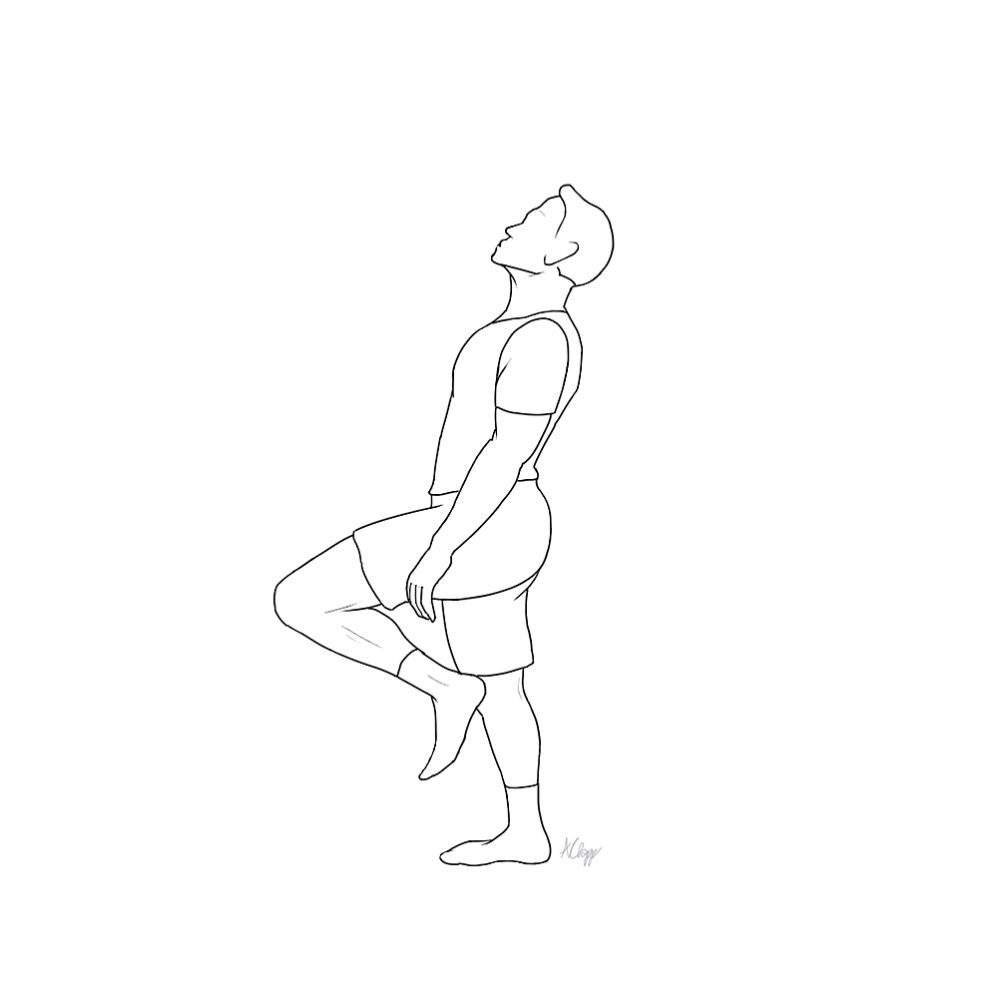
Stork test for spondylosis. The patient stands on one leg and performs lumbar extension. This is then repeated with the other leg. The test is positive if there is ipsilateral pain during extension, which indicates lumbar joint pathology such as spondylosis, spondylolisthesis, or a limbus vertebra. The figure is original and was designed for this article.

A review of systems did not reveal any other pertinent findings. On examination, the patient showed normal responses to the straight leg raise test and no neurologic signs. His range of motion was normal with extension and flexion of the lumbar spine, but associated pain occurred over the paravertebral muscles. Palpation of the proximal lumbar spine vertebrae revealed mild tenderness. Paraspinal spasms were noted. No signs suggestive of inflammation were evident. The stork test caused mild pain over the lower back (Figure 2).

Conservative treatment was initiated which included rest for two weeks and icing of the painful area of his back for 15-20 minutes a day, up to three times a day. The patient was also advised to avoid any activities and positions that might lead to back pain. He was advised to take ibuprofen as needed for pain and cyclobenzaprine as needed for spasms. He was referred to physical therapy for local modalities and core-strengthening exercises. His back pain had resolved by the two-week follow-up. Approximately three weeks later, he was pain-free, even during activities.

Diagnosis

**Figure 3:**
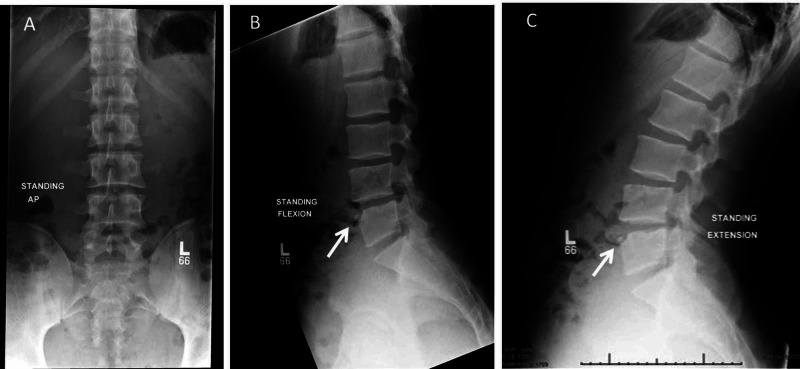
Anteroposterior view of the lumbar spine of the patient showing no acute abnormalities (A). Lateral views of the lumbar spine of the patient; white arrows indicate a well-corticated triangular osseous focus at the anterosuperior aspect of the L5 vertebral body, seen on the lumbar spine flexion view (B) and extension view (C). This is most consistent with a limbus vertebra at L5.

In this athlete, treatment was geared towards mechanical back pain and spasms, and it appeared to be effective. The patient had expressed desire at the time of the consultation to participate as soon as possible in a college-level volleyball competition. The lengthy symptomatology (three weeks) in this adolescent collegiate athlete who performed repetitive hyperextension as a volleyball player who was planning to participate in an upcoming competition, combined with the physical examination findings and positive stork test, indicated a need for radiography due to increased suspicion of spondylolysis [10-12]. Anteroposterior view of the lumbar spine was normal (Figure 3A). Lateral radiography views of the lumbar spine in flexion and extension views (Figures 3B, 3C) showed a well-corticated triangular osseous focus at the anterosuperior aspect of the L5 vertebral body. These findings are classic for a limbus vertebra at L5 and no further workup was required.

A single-photon emission computerized tomography scan or magnetic resonance imaging would have been an appropriate next step had spondylosis been noted on radiography or had the patient not responded to conservative treatments [10-12].

## Discussion

Athletes often present in primary care settings with lower back pain. When they have a history and physical examination findings consistent with mechanical back pain, conservative treatment (e.g., nonsteroidal anti-inflammatory drugs, muscle relaxants, rest, and ice) is appropriate. Most adolescent patients with acute lower back pain need not undergo radiography particularly with concern for radiation exposure, although it may be reasonable for athletes who perform repetitive extension, present with a prolonged duration of pain, have spinal point tenderness, or have a sports-related decision to make (e.g., participation in a competition). The differential diagnoses for lower back pain in an adolescent athlete who are most commonly recognized in the literature are listed in Table 1. It is important to consider limbus vertebra in the differential.

**Table 1:** Differential diagnoses for back pain in an adolescent athlete (arranged from most to least common; adapted from data reported by Micheli et al., 1995 [13]). ^a^Limbus vertebra is not uncommon and is an important differential diagnosis for back pain in adolescents, although there are no current studies to quantify the incidence [5].

Rank		Low back pain etiology
1		Spondylolysis/spondylolisthesis
2		Hyperlordotic mechanical back pain
3		Discogenic
	3a	Herniated
	3b	Degenerated
		Both
4		Scoliosis
5		Lumbosacral strain
6		Hamstring strain
		Trochanteric bursitis
7		Osteoarthritis
		Spinal stenosis
		Neoplasm
		Ankylosing spondylitis
		Limbus vertebra^a^

The vertebral ring apophysis ossifies between six and 13 years of age and fuses with the vertebral body during skeletal maturation at around 18 years of age [14]. Before fusion of the physis occurs, herniation can arise at a weak point between the ring apophysis and the adjacent vertebral body, resulting in an anterior or posterior limbus vertebra [15]. This can often be visualized with radiographic imaging. Computed tomography is considered the best method for further examination for uncertain diagnoses [2].

A review of the literature revealed that a limbus vertebra finding has been reported to cause mechanical lower back pain [5-8]. The underlying mechanism for this lower back pain has been described as spasms of the muscle surrounding the abnormality and, in some cases, radicular pain [5,8]. Limbus vertebra can be an incidental finding and asymptomatic patients usually require no medication or treatment [1]. If a limbus vertebra is found in a symptomatic individual, the symptoms may or may not stem from the presence of the limbus vertebra; however, the approach taken in our volleyball player case was to address it as the likely cause of lower back pain and to initiate conservative treatment with close follow-up until the pain resolved.

Clinicians may be concerned with radiographic images showing an unusual triangular osseous focal area that is an intraosseous herniation of the nucleus pulposus which may prompt further imaging (e.g., computed tomography, magnetic resonance imaging, bone scans, etc.). Clinicians need to be familiar with a limbus vertebra diagnosis and the appropriate conservative treatment plan so that further imaging, stronger medications, or seeking specialist consultations are not immediately ordered, particularly in asymptomatic individuals, to avoid unnecessary expenses to the patient.

## Conclusions

Limbus vertebra is a condition that should be part of the differential diagnosis for uncomplicated lower back pain. Familiarity with this diagnosis may help primary care physicians reduce unnecessary radiologic testing and cost to the patient.
